# Intra-thymic bronchogenic cyst an extremely rare tumor of anterior mediastinum in adults

**DOI:** 10.1186/s13019-018-0809-3

**Published:** 2018-11-20

**Authors:** Shadi Hamouri, Muad Hatamleh, Jamal Alaydi, Hani Alhadidi, Mazen Alomari, Najla Aldaoud, Bashar Darayseh

**Affiliations:** 1 Department of General Surgery and Urology, Faculty of Medicine, Jordan University of Science and Technology, Irbid, 22110 Jordan; 2 Division of Thoracic Surgery, Department of Surgery, King Hussein Medical Center, Jordanian Royal Medical Services, Amman, Jordan; 3 Department of Pathology and Microbiology, Faculty of Medicine, Jordan University of Science and Technology, Irbid, Jordan

**Keywords:** Anterior mediastinal lesions, Intra-thymic cyst, Bronchogenic cyst, VATS thymectomy

## Abstract

**Background:**

Intra-thymic bronchogenic cysts are a rare entity but should be considered in the differential of all non-invasive thymic masses.

**Case presentation:**

We describe a 50-year-old patient who was found to have an incidental thymic mass on computer tomography of the chest. Non-invasive thymoma was suspected and a thoracoscopic thymectomy was performed. Final pathology revealed a bronchogenic cyst.

**Conclusion:**

Intra-thymic bronchogenic cysts are extremely rare tumors of the anterior mediastinum. It should be considered in differential diagnosis of anterior mediastinal masses.

## Background

Benign thymic pathologies are rare and they are not discussed fully in the published literature. One of these pathologies is intrathymic cysts that can be either congenital or acquired. Intrathymic bronchogenic cyst is an extremely rare congenital cystic thymic pathology. Bronchogenic cysts are rare congenital anomalies that develop due to a foregut abnormal budding. It is located either in the mediastinum namely the middle and posterior mediastinum or in the lung parenchyma [1]. Anterior mediastinal bronchogenic cysts are rare and intrathymic bronchogenic cyst are extremely rare [2].

Herein we present a case in which an asymptomatic intrathymic bronchogenic cyst was discovered incidentally on the CT scan of the chest while evaluating a subcentimetric pulmonary nodule.

## Case presentation

**Fig. 1:**
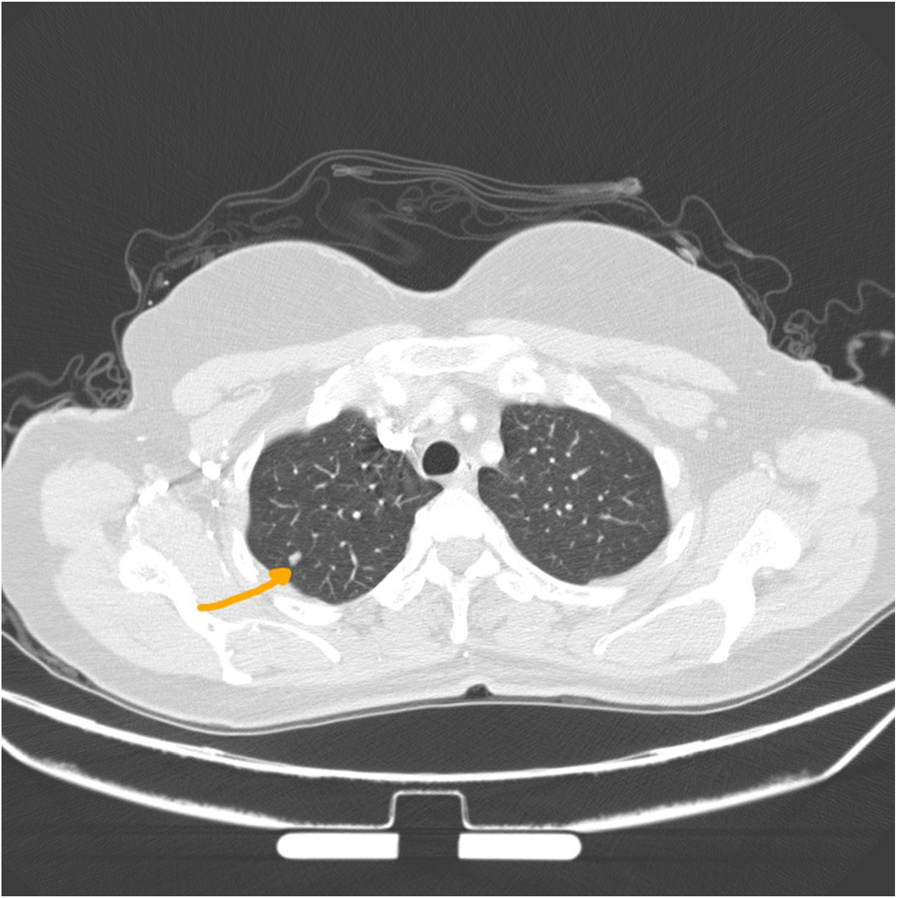
CT scan of the chest showing the small pulmonary nodule in the right upper lobe

**Fig. 2:**
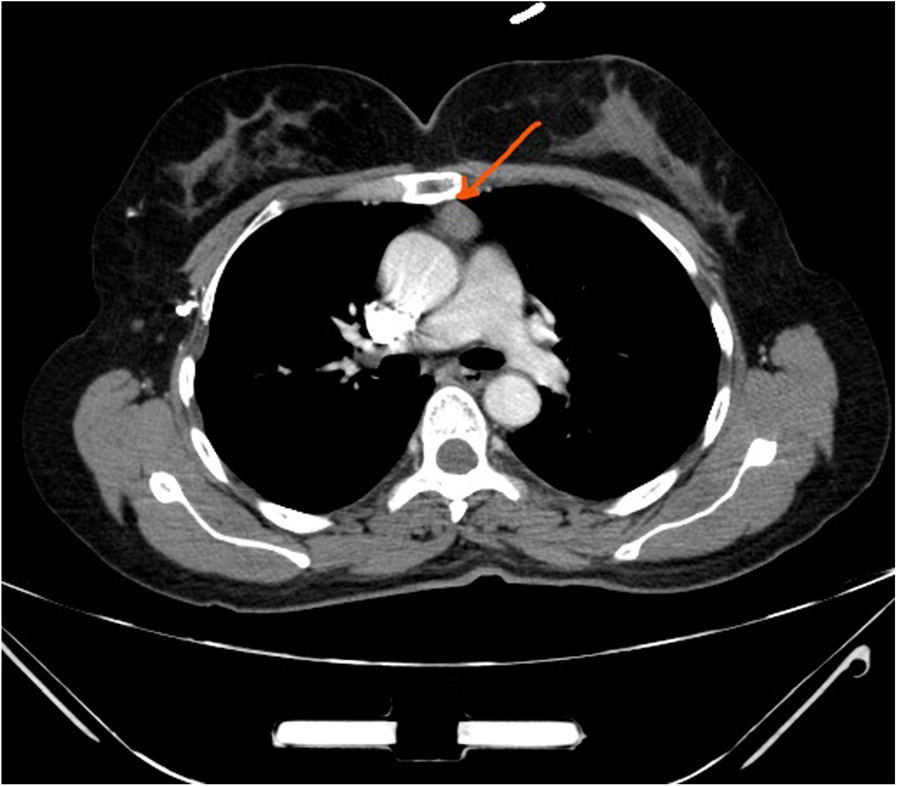
CT scan of the chest showing a 2 × 2 cm well-defined oval shaped enhancing soft tissue anterior mediastinal tumor with (Hounsfield units of 55)

**Fig. 3:**
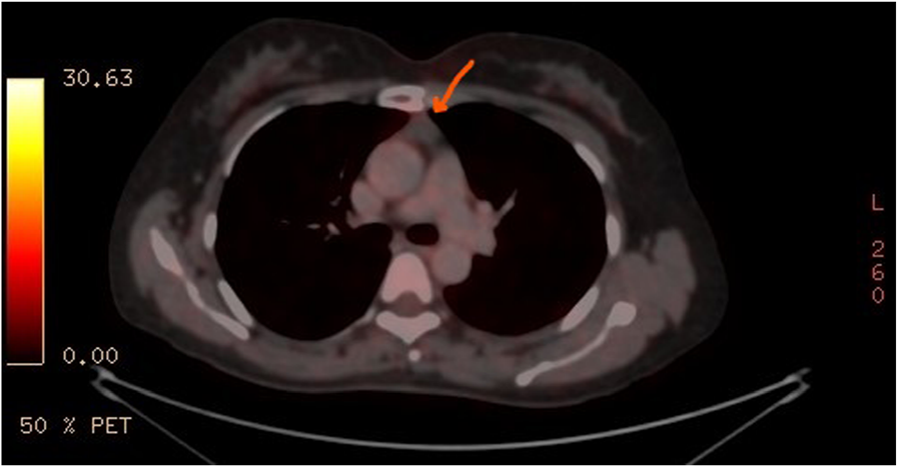
Integrated PET/CT demonstrating that the mediastinal lesion is not avid for FDG

A 50-year-old female patient with a previous history of hypothyroidism and no past surgical history was transferred from Ear, Nose and Throat (ENT) outpatient clinic for further evaluation of a 7 mm solitary pulmonary nodule in the right upper lobe [Fig. 1]. This was incidentally discovered in a computer tomography (CT) scan of the neck done for evaluation of upper respiratory tract symptoms. A CT of the chest was done to assess the rest of the lung parenchyma. In addition to the previously mentioned peripherally located nodule, the (CT) showed a 2 X 2 cm well-defined oval shaped enhancing soft tissue anterior mediastinal tumor with (Hounsfield units of 55). All radiological findings were suggestive of thymoma [Fig. 2]. As the pulmonary nodule has an intermediate risk for malignancy an integrated positron emission tomography with computer tomography (PET/CT) was performed. Nor the nodule neither the mediastinal lesion showed any hypermetabolic activity [Fig. 3] so watchful waiting was elected for the management of the nodule. Due to the age of the patient and the CT radiological manifestations including the size of the mediastinal lesion, absence of intralesional fat, loss of triangular thymic shape, a soft tissue Hounsfield units as well as the oval shape of the lesion, the likelihood of epithelial thymic tumor namely thymoma has increased [3]. These clinical and radiological characteristics in addition to the patient’s wish encouraged us for the option of surgical resection rather than watchful waiting policy. Based on the high index of suspicion of non-invasive thymoma a right sided video-assissted thoracoscopic thymectomy. The patient was placed in left lateral decubitus position. A roll was placed under the patient’s side, elevating the body by approximately 30 to 45 degrees. Three thoracoscopic ports were used with insufflation of pleural space with CO_2_ (flow of 4–6 l/m, pressure of 3–5 mmHg) was performed. After assessment of the whole chest cavity, complete thymectomy including the above-mentioned tumor as well as the adjacent mediastinal fat was performed. The specimen was retrieved and a 24-Fr chest drain was inserted. A digital palpation through the thoracoscopic incisions could not detect the nodule so resection was not performed.

The post-operative course was uneventful and the patient was discharged home after chest tube removal on the first postoperative day.

**Fig. 4:**
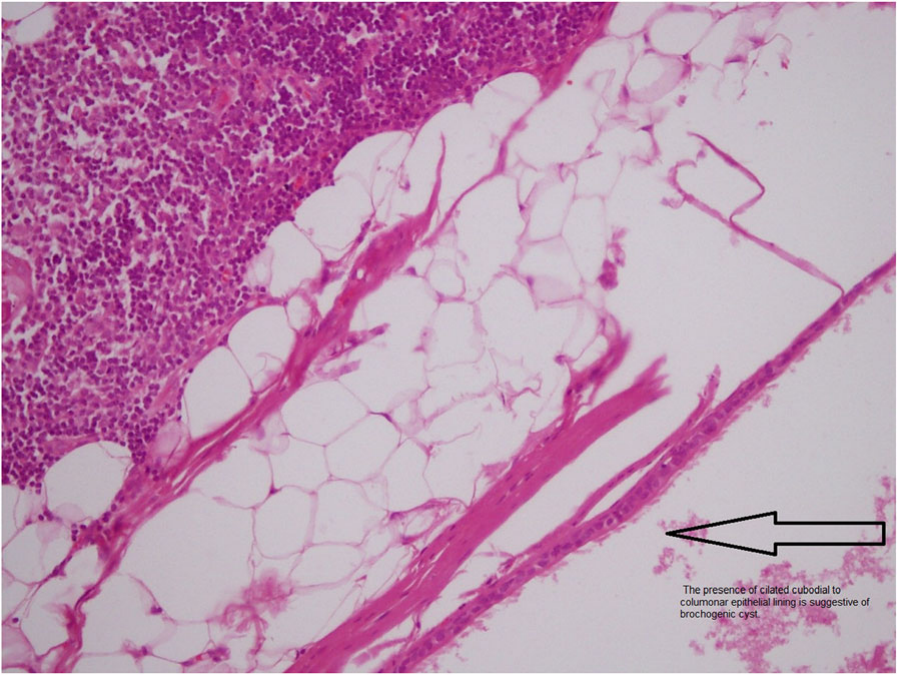
Photomicrograph of intrathymic bronchogenic cyst showing a columnar ciliated epithelium

The histological examination revealed grossly a completely excised thymus that contained a unilocular cyst filled with white creamy material. Histologically the cyst was lined with ciliated columnar epithelium resembling respiratory epithelium that supported the diagnosis of intrathymic bronchogenic cyst [Fig. 4].

## Discussion

Benign thymic pathologies such as hyperplasia or thymic cysts are rarely studied. In the published literature there are few studies focused on the clinical and radiological manifestations of benign entities of the thymus. Usually they are grouped as benign thymic disorders [4].

Thymic cysts are relatively rare pathology that can be seen in any age group. They represent 1–3% of all mediastinal masses [4]. They may be congenital or acquired [4, 5]. The congenital cyst can be found anywhere along the thymopharyngeal duct [5]. They usually unilocular, have a thin wall, contain clear fluid and appears in the pediatric age group [4]. The acquired cyst, may develop after chemotherapy, radiotherapy, HIV infection, thoracotomy, or associated with thymic tumors that disrupt and compress normal thymic tissue. [4, 5]. These are multilocular contain proteinaceous or hemorrhagic material which results in soft-tissue attenuation of the mass and can create a diagnostic dilemma [5].

The most common mediastinal cysts are the bronchogenic cyst, which usually occurs in the posterior and middle mediastinum. They account for 10 to 15% of all primary mediastinal masses [1]. The identification of bronchogenic cyst has increased because of more advanced diagnostic methods, and regular medical checkups were performed in recent years [6]. The site of the bronchogenic cyst determined by the embryological period of growth at which the abnormality occurs. When the abnormal buddings happens in the course of the early growth, the cyst apt to be located along the tracheobronchial tree. Cysts that developed later are more peripheral and may be sited within the lung parenchyma. [7]. The majority of the cysts are mediastinal bronchogenic cysts and closely related to the tracheobronchial tree near the tracheal carina [1, 4, 7].

Infrequently, the cysts have occurred in rare sites, including skin and subcutaneous tissues, neck, pericardium, diaphragm, mesentery of the bowel, and the intramedullary part of the spine. They have also been reported to extend from the mediastinum through the diaphragm into the abdomen as dumbbell cysts [7].

Rarely, bronchogenic cyst can occur within the thymus gland. The first reported case was published by Taniwaki and colleagues in 1997 [2]. After which few cases were also reported. These cysts are asymptomatic and discovered incidentally in imaging studies done for another reasons. Usually they are of very little clinical importance, and can go undetected except if they produce compressive symptoms [4, 8]. Multi-slice CT is used to delineate the cyst shape, size, location, attenuation and relation to the adjacent structures. Nevertheless, typical imaging characteristics can only be seen in 10–40% of patients [6]. Soft-tissue attenuation on chest CT caused by protein-rich cyst fluid can make the preoperative differentiation from solid neoplasm mainly thymoma very difficult. This has been reported in 40–63% of the reported cases [4, 6]. MRI can be beneficial in demonstrating the cystic nature of what appeared to have a soft-tissue attenuation on the CT [9]. Unless there is no accompanied inflammation in the bronchogenic cyst or underlying intrathymic pathology like hyperplasia or invasive tumor the intrathymic bronchogenic cyst has no fludeoxyglucose (FDG) uptake on the (PET/CT) [10].

The definitive diagnosis of intrathymic bronchogenic cyst is usually made after complete excision of the lesion by illustration of the histological characteristics of the bronchogenic cyst like ciliated columnar epithelium with or without infiltration by chronic inflammatory cells, presence of cartilage, smooth muscle, bronchial glands and nerve tissue [1].

## Conclusion

Intra-thymic bronchogenic cysts are extremely rare tumors of the anterior mediastinum. The overlapping radiological characteristics with solid tumors, especially on the CT makes the preoperative radiological diagnosis very difficult. It should be considered in differential diagnosis of anterior mediastinal masses. The final diagnosis would be given after surgical excision and histopathological examination.

## Abbreviations

CT: Computer tomography
ENT: Ear, Nose and Throat
FDG: Fludeoxyglucose
Fr: French
PET/CT: Positron emission tomography with computer tomography
VATS: Video-assisted thoracoscopic surgery
